# Challenging convention: primary ureteroscopy for stone disease with concomitant infection—a multicentre study

**DOI:** 10.1007/s11845-026-04291-5

**Published:** 2026-03-16

**Authors:** James Connor, Steven Anderson, John O’Kelly, Karl Ringrose, Ailish Naughton, David Galvin, Kieran Breen, Diarmaid Moran, Mark Quinlan, Barry McGuire, Niall Davis

**Affiliations:** 1 Department of Transplant, Urology and Nephrology, Beaumont Hospital, Dublin, Ireland; 2 Department of Urology, St Vincent’s University Hospital, Dublin, Ireland; 3 Department of Surgery, Royal College of Surgeons in Ireland, 123 St Stephens Green, Dublin, Ireland; 4 Department of Urology, Beaumont Hospital and Royal College of Surgeons in Ireland, Dublin, Ireland

**Keywords:** Endourology, Laser lithotripsy, Urolithiasis, Urosepsis

## Abstract

**Introduction:**

Obstructing urolithiasis with concomitant infection presents a major clinical challenge. While urgent decompression is standard, the safety of early definitive stone treatment remains uncertain. This study evaluates the outcomes of primary ureteroscopy compared with delayed intervention following decompression.

**Objectives:**

To investigate whether early ureteroscopy is a safe treatment option for the management of urolithiasis in the context of concomitant urinary infection.

**Methods:**

A multi-institutional observational study (2019–2023) included adults with radiologically confirmed obstructing urolithiasis and infection. Patients underwent either primary ureteroscopy within 24 h without prior decompression (Group A) or decompression with deferred ureteroscopy during the same admission (Group B). Primary outcomes were postoperative complications and stone-free rate (SFR). Secondary outcome was hospital length of stay (LOS). Statistics were performed using odds ratios (OR) or mean difference and comparative parameters.

**Results:**

Forty-four patients were included: 29 in Group A and 15 in Group B. Group A patients had lower ASA scores and smaller mean stone size (7.2 mm vs 11.2 mm, p = 0.003). The overall SFR was 79.6%, significantly higher in Group A than Group B (89.7% vs 60%, OR 5.78, p = 0.029). Median LOS was shorter in Group A (3 vs 8 days, p = 0.038). Postoperative complication rates were low and comparable. Prolonged infection occurred in 6% of Group A and 13% of Group B (p = 0.48).

**Conclusion:**

Primary ureteroscopy may be a safe and effective strategy in selected patients with obstructing urolithiasis and concomitant infection, offering reduced hospitalisation without increased complications. Larger prospective studies are required to validate these findings.

## Introduction

Obstructing urolithiasis complicated by a concomitant urinary tract infection (UTI) presents a clinical challenge. The development of sepsis in such cases is associated with significant morbidity and potentially life-threatening conditions if not managed urgently and appropriately [1, 2]. The primary focus of intervention is urgent decompression of the obstructed kidney combined with appropriate antimicrobial therapy. Decompression can be achieved by either inserting a ureteral stent or a percutaneous nephrostomy (PCN), while definitive treatment of the stone is typically deferred [3]. However, the optimal timing of definitive stone treatment remains unclear. While interval decompression followed by elective surgery is widely practiced, the role of early definitive intervention, such as ureteroscopy with stone removal, has not been clearly defined, especially in the context of active infection [4].

In recent years, ureteroscopy has evolved into a standard minimally invasive technique for treating ureteral stones, with laser lithotripsy being the preferred method of fragmenting the stone. It offers the advantage of stone clearance during the initial procedure, potentially reducing the need for subsequent interventions. However, concerns remain regarding the safety of performing ureteroscopy in patients with concomitant infection, as manipulation of the urinary tract during active infection could worsen the infection or lead to other complications [5]. Nonetheless, a growing body of evidence suggests that in carefully selected patients, primary ureteroscopy with laser lithotripsy may offer a safe and effective alternative to deferred intervention [6]. Furthermore, early definitive treatment offers the potential to reduce hospital length of stay, ureteral stent dwell time and the need for multiple procedures.

This observational study was undertaken to assess the outcomes of primary and early post-decompression ureteroscopy and definitive stone treatment, in patients presenting with obstructing urolithiasis complicated by infection.

## Methods

### Study design and population

This study was designed as a multi-institutional observational study, of prospectively collected data, conducted to evaluate the safety and efficacy of primary ureteroscopy with definitive stone treatment in patients presenting with obstructing urolithiasis and consequent infection. The study followed the STROBE guidelines for observational studies [7]. Patients were enrolled between 2019 and 2023, with the inclusion criteria requiring all participants to have a radiologically confirmed diagnosis of obstructing urolithiasis with superimposed infection.

### Inclusion criteria

Eligible patients were adults (age ≥ 18 years) who presented to the emergency department with symptomatic obstructing urolithiasis and concomitant urosepsis. Only patients who presented acutely with a new episode of colic and computed tomography (CT) evidence of a ureteral calculus resulting in hydronephrosis were considered for inclusion. For the purposes of this study, urosepsis was defined according to the Third International Consensus Definitions for Sepsis and Septic Shock (Sepsis-3) [8]. Specifically, patients were considered to have urosepsis if they presented with an infection-induced systemic inflammatory response in the setting of confirmed urolithiasis, characterised by the presence of two or more of the following clinical features: pyrexia (> 38 °C) or hypothermia (< 36 °C), tachycardia (> 90 beats/min), leukocytosis (> 12,000 cells/µL) or leukopenia (< 4,000 cells/µL), and elevated (> 50 mg/l) serum C-reactive protein (CRP) or other markers of infection, without evidence of an alternative source of infection.

### Exclusion criteria

Exclusion criteria included patients with hemodynamic instability requiring immediate intensive care unit (ICU) admission, those presenting after failed expectant management of urolithiasis, patients with an active urological malignancy, and those with contraindications to ureteroscopy or general anaesthesia.

### Intervention and operative technique

All patients were initially resuscitated with intravenous fluids, analgesia, anti-emetics and commenced on empiric intravenous antimicrobials (initially co-amoxiclav and gentamicin unless contra-indicated). Patients were then subdivided into two groups: Patients who were deemed to be clinically stable by the operating urologist and anaesthetist, and had ureteral stones that were felt to be amenable to primary ureteroscopy without an initial period of decompression within 24 h of presentation (Group A); Patients who were felt to be unstable and therefore underwent urgent decompression via the insertion of either a percutaneous nephrostomy or ureteral stent, with definitive treatment deferred until clinically stable but during the same hospital admission (Group B).

In all cases, ureteroscopy was performed using either a semi-rigid or flexible ureteroscope to achieve definitive stone treatment. All rigid ureteroscopies were performed using a 6/7.5Fr semi-rigid ureteroscope (Richard Wolf GmbH™, Knittlingen, Germany), while all flexible ureteroscopies were performed using a single-use 7.7/9.5Fr flexible ureteroscope (LithoVue™, Boston Scientific™, Massachusetts, United States of America). Fluoroscopy was used in all cases and patient imaging was available in theatre. There was limited use of contrast to perform retrograde pyelography, and the renal pelvis was typically maximally aspirated at the start of each case, with the subsequent urine sent for microscopy, culture and sensitivity testing. Ureteral stones were fragmented using a 270 µm laser fibre and either a 30 W holmium: yttrium–aluminum-garnet (Ho:YAG) laser (Auriga™ 30, Boston Scientific™, Massachusetts, United States of America) or a 35 W Ho:YAG laser (Dornier Medilas® H Solvo® 35, Dornier MedTech Laser GmbH, Wessling, Germany) typically using settings of 0.5–1.0 J and 6–8 Hz. A 36 or 46 cm, 11/13Fr ureteral access sheath (Navigator™, Boston Scientific™, Massachusetts, United States of America) was used during all flexible ureteroscopies, and no pressure irrigation devices were used. Stone fragments were basketed and removed if deemed necessary by the operating surgeon. All cases were performed by consultant level surgeons. Regular communication was maintained with the consultant anaesthetist to ensure the patient remained haemodynamically stable during the procedure, and a 6Frx24cm or 6Frx26cm stent was inserted at the end of every case, with a date for removal planned at the discretion of the operating surgeon.

### Patient demographics and clinical characteristics

Data on patient demographics and clinical characteristics were collected, including age, gender, comorbidities (such as diabetes, chronic kidney disease, and cardiovascular disease), body mass index (BMI), a history of recurrent urolithiasis, and the presence of urinary abnormalities. Stone characteristics were documented, including size and location. Clinical parameters at the time of presentation, including pyrexia, white blood cell (WBC) count, and CRP levels, were recorded, as well as results of any blood or urine cultures performed.

### Outcomes

The primary outcomes included the incidence of postoperative complications, particularly refractory infection as defined by worsening of clinical parameters such as haemodynamic instability, need for ICU, escalation of antibiotics, or requirement for reintervention. Complications were categorised according to the Clavien Dindo system [9]. Stone-free status was also measured and was defined as the absence of symptomatic and detectable stone fragments on postoperative imaging (ultrasound or non-contrast computed tomography) within 30 days after surgery. The secondary outcome of the study was hospital length of stay (LOS) measured in days.

### Statistical analysis

Descriptive statistics were used to summarize baseline characteristics, clinical parameters, and outcomes. Continuous variables were reported as means and standard deviations as appropriate. Categorical variables were expressed as frequencies and percentages. The outcomes of SFR and LOS were compared between groups using odds ratio (OR) with Fisher’s exact testing, and Mann Whitney U testing of difference in medians as appropriate respectively. A p-value of < 0.05 was considered statistically significant. All analyses were conducted using STATA Statistical Software (STATA v17, College Station, Tx: StataCorp LLC).

### Ethical considerations

The study was conducted in accordance with the ethical standards of the institutional and/or national research committee and with the 1964 Helsinki Declaration and its later amendments or comparable ethical standards. Ethical approval was also given by local ethical authorities (ID CA2024/182).

## Results

### Patient characteristics

Forty-four patients underwent early ureteroscopy and laser lithotripsy for septic urolithiasis and were suitable for inclusion in our analysis. 29 patients underwent primary ureteroscopy and laser lithotripsy (Group A) and 15 patients underwent decompression prior to stone removal (Group B). Patient demographics and clinical parameters for the two groups are summarised in Table 1. Participants in Group A had notably lower ASA scores with 96.6% being ASA I/II and 3.4% being ASA III, compared to Group B where 53.4% were ASA I/II and 46.6% were ASA III. The overall median stone size was 7 mm (range 3–21 mm). Group B had a significantly larger stone burden with a mean stone size of 11.17 mm (range 6–21 mm) compared to Group A with a mean of 7.18 mm (range 3–10 mm) *p* = 0.003. The majority of stones were located proximally (66%, n = 29). Causative pathogens on urine culture were many and varied between groups (Table 1). Group B had a significantly higher CRP (247.6 ± 144.2) compared to Group A (123.8 ± 114.8) p = 0.003. SFR, LOS and post operative complications for group A and B are displayed in Table 2.

**Table 1:** Patient characteristics

Variable	Group A	Group B	p
	n = 29	n = 15	
Age (years), mean (range)	54.5(± 17.7)	65.9(± 18.8)	0.054^a^
Gender, n (%)			
*Female*	15 (51.7)	7 (46.6)	
*Male*	14 (48.3)	8 (53.3)	1^b^
ASA score, n (%)			
*I*	12 (41.4)	2 (13.4)	
*II*	16 (55.2)	6 (40)	0.089^b^
*III*	1 (3.4)	7 (46.6)	0.526^b^
*IV*	0	0	0.001^b^
Stone size, n (%)			
> *10 mm*	2 (6.9)	7 (46.6)	
*5–10 mm*	24 (82.8)	7 (46.6)	
< *5 mm*	3 (10.3)	1 (6.8)	0.004^b^
*Stone size, mean (SD)*	7.18 (2.98)	11.17 (5.30)	
Location, n (%)			0.019^b^
*Proximal*	16 (55.2)	13 (86.7)	
*Mid*	2 (6.9)	1 (6.7)	1^b^
*Distal*	11 (37.9)	1 (6.7)	0.003^a^
Febrile, n (%)	20 (68.9)	11 (73.5)	
Hypotensive, n (%)	5 (17.2)	6 (40)	
White cell count × 10^9^/L, mean (SD)	16.2 (± 5.2)	16.2 (± 4.2)	0.048^b^
C reactive protein, mg/dL, mean (SD)	123.8 (± 114.8)	247.6 (± 144.2)	
Urine Culture, n (%)			1^b^
*Escheria Coli*	4 (13.7)	5 (30.0)	0.035^b^
*Klebsiella Pneumoniae*	0	2 (13.4)	1^b^
*Enterococcus*	4 (13.7)	1 (6.7)	0.145^b^
*Proteus mirabalis*	1 (3.4)	2 (13.4)	1^a^
*Serratia sp*	1 (3.4)	0	0.003^a^
*Mixed growth*	9 (31.1)	2 (13.4)	
*Negative*	5 (17.2)	1 (6.7)	
*Not available*	5 (17.2)	2 (13.4)	

*SD standard deviation,* p values of differences by ^a^independent T test and ^b^Fisher’s exact test

**Table 2:** Operative results and differences between groups with odds ratios and p values of differences by independent t test as appropriate. SFR = stone free rate. LOS = length of hospital stay, CI = confidence interval

	Group A (n = 29)	Group B (n = 15)	Odds ratio (95% CI)	*p*
Mean time to theatre (days)	1 (0–1)	5 (3–14)		
SFR (%)	89.7	60	5.78, (1.19, 28.04)	0.029
Mean LOS	3 (1–19)	8 (3–45)		0.038
Clavien Dindo complications	2	2	0.48 (0.06, 3.81)	0.48

### Post-operative complications

Group A had two Clavien-Dindo grade II complications, with two patients developing prolonged post operative urinary tract infections who had an ASA grade of II and III. One of those had significant comorbidities of a stroke with left hemiparesis and type 2 diabetes. Both patients responded to antibiotic therapy without requirement for additional acute surgical or radiological intervention.

Similarly, there were two Clavien-Dindo grade II complications in group B, both of whom were ASA grade III. Two patients developed postoperative urinary tract infections. These cases involved a 73-year-old male and 59-year-old female who both had a background of cerebrovascular accident (CVA), hemiparesis and long-term catheter and had a large stone burden. Both cases responded to intravenous antibiotics. There was no statistically significant difference in the rate of post-operative complications between the two groups (OR 0.48, 95% CI 0.06, 3.81, p = 0.48). However, post hoc analysis revealed a statistical power of 12.4%.

### Stone free rate

In group A, all patients underwent treatment within 24 h of admission to hospital, and complete stone clearance was achieved in 89.7% of cases (n = 26). One patient (3.4%) developed post-operative pyrexia which was managed at ward level with IV fluids and continued antibiotics. In group B, patients had a period of renal decompression prior to definitive stone treatment. Ten patients (67%) underwent cystoscopic insertion of a ureteral stent while the remaining 5 patients (33%) underwent insertion of a percutaneous nephrostomy, 2 of whom received concomitant antegrade stenting. Complete stone clearance was achieved in 60% of patients (n = 9) in Group B. There was a statistically significant higher SFR in patients who underwent primary ureteroscopy compared to those who had deferred treatment following a period of decompression (OR 5.78, 95% CI 1.19, 28.04, p = 0.029). Fisher’s exact test was also performed, confirming a statistically significant result (test statistic = 0.0439, p < 0.05). Post hoc analysis generated a statistical power of 62.9% to detect significant differences between groups.

### Duration of inpatient stay

In group A, the median LOS was 3 days (range 1–19). In group B, the median length of time from drainage to ureteroscopy was 5 days (range 3–14), and the median length of stay was 8 days (range 3–45). The LOS was significantly shorter for patients in Group A compared to those in Group B (U = 99, Z = −1.77, p = 0.038) with a post hoc analysis of statistical power of 88.1%.

### Discussion

The need for urgent antimicrobial treatment and upper tract decompression in patients presenting with infection secondary to obstructing urolithiasis is well established. The optimum timing of definitive stone treatment however remains controversial. Historically, such patients were managed by deferred ureteroscopy following a prolonged course of antimicrobial therapy, typically during a second hospital admission [10]. While this approach allows for the resolution of the initial infection prior to ureteroscopic treatment, it is not without risk. Multiple studies have demonstrated that ureteral stent dwell times of greater than 30 days is associated with an increased risk of emergency department presentation and post-operative infection [11–13]. These factors, coupled with the need for further hospital admissions with deferred treatment are important clinical and economic considerations. It is therefore unsurprising that there has been a recent change in the approach to treating sepsis secondary obstructing urolithiasis, with a growing body of evidence to support earlier definitive treatment.

One randomised controlled trial (RCT) by Astroza et al. in 26 patients evaluated the safety of early ureteroscopic treatment (EUT) following initial decompression in patients presenting with urosepsis secondary to ureteral calculi. Patients were randomised to undergo EUT, defined as ureteroscopy and attempted stone clearance during their initial hospital presentation with sepsis, or deferred treatment in which they were discharged and brought back electively for definitive treatment. The authors found no differences in complication rates between the two groups, and shorter stent dwell times in the EUT group [14]. These findings are supported by the low complication rate and high stone clearance rate seen in our study, highlighting that these patients can be safely managed during their initial hospital admission.

The role of primary ureteroscopy without an initial period of decompression in this patient cohort remains poorly investigated but offers obvious advantages in that patients can potentially be treated without requiring multiple invasive procedures and with shorter hospital stays. The results of our study support this hypothesis as the SFR in Group A was 89.7%. This is consistent with the results of a RCT by Bakr et al. in which 124 patients with distal ureteral stones and “mild sepsis” were randomised to either primary ureteroscopy or ureteral stent insertion and deferred definitive treatment, and reported a SFR of 98.1% in patients who underwent primary ureteroscopy without an increase in adverse events [15]. The slightly lower SFR seen in our study is likely explained by the fact that stones located in any segment of the ureter were included in our study, with the majority of stones located in the proximal ureter, as opposed to the inclusions of distal stones only in the study by Bakr. et al.

A systematic review and meta-analysis by Alsawi et al. noted that delayed ureteroscopy after decompression was associated with a comparable SFR and reintervention rate compared to primary ureteroscopy [16]. Conversely, in our study, patients who had delayed stone treatment after an initial period of decompression had a notably lower SFR than those who underwent primary ureteroscopy (60% vs 89.7% respectively). This likely reflects the increased complexity of patients requiring delayed intervention, including those with larger stone burdens, multiple comorbidities, or more prolonged infections. However, it is also possible that delayed ureteroscopy is a more challenging procedure when performed in the first weeks following initial decompression due to increased ureteral oedema and inflammation. This highlights the potential benefits of addressing both the stone and infection simultaneously through primary ureteroscopy, which may reduce the need for subsequent procedures and lower the overall treatment burden. Finally, it is important to note that the patient cohort in the study by Alsawi et al. is different to the cohort in our study, as our study is exclusively on patients presenting with infective symptoms, and the average stent dwell time in those who received initial decompression was significantly shorter.

The low rate of postoperative complications in Group A is encouraging and is consistent with the growing consensus that primary ureteroscopy can be safely performed in selected patients with obstructing stones and urinary infection [15, 16]. Multiple studies have corroborated these results, demonstrating that early intervention in stable patients does not significantly increase the risk of postoperative sepsis or other serious complications [14, 17]. However, the higher rate of complications in Group B, where two patients developed postoperative sepsis, emphasises the importance of patient selection and timing in managing these cases. Patients who experienced postoperative sepsis frequently had substantial comorbidities, such as a history of cerebrovascular accidents, hemiparesis, and long-term catheter use, highlighting the challenges of treating more complex patients.

The median LOS in Group A (3 days) was significantly shorter than in Group B (8 days), reflecting the potential benefits of early stone removal in reducing hospitalisation times. Shorter hospital stays minimise the risk of nosocomial infections, which are particularly concerning in septic patients. The longer LOS in Group B reflects the more complicated clinical course often seen in patients undergoing delayed ureteroscopy. These patients, who required a period of decompression before definitive stone removal, likely experienced prolonged recovery times due to the need for continued infection control and the management of comorbid conditions. The overall LOS in both groups is in keeping with a previous study of 56 participants, which showed a mean LOS of 7 days [6].

This study highlights the importance of tailored decision-making in the management of obstructing urolithiasis complicated by infection. The high stone clearance rates and low complication rates observed in Group A suggest that primary ureteroscopy is a safe and effective option for haemodynamically stable patients with concomitant infection and obstructing urolithiasis. However, it is essential to recognise the role of patient selection in determining outcomes. Patients with significant comorbidities, larger stone burdens, or more severe infections may benefit from a more conservative approach, including delayed stone removal following decompression and infection control.

**Fig. 1:**
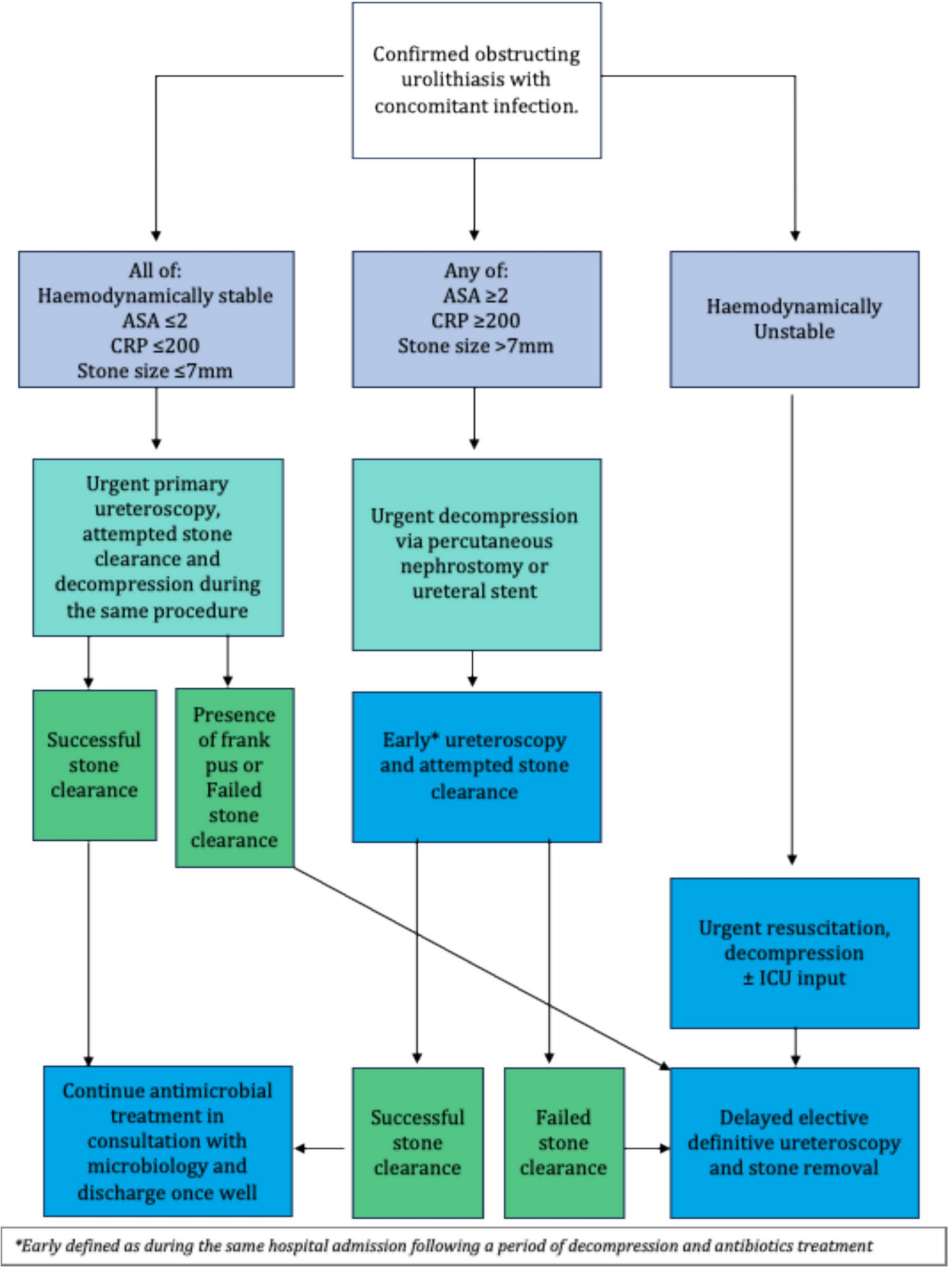
Proposed algorithm for management of patients presenting to ED with urolithiasis and concomitant urinary infection

We propose a clinical algorithm to guide the timing of definitive stone removal in patients with obstructive urolithiasis and concomitant infection for future clinical trials (Fig. 1). Initial stratification is based on the patient’s hemodynamic stability, with unstable patients or those requiring intensive care undergoing urgent decompression with delayed definitive stone removal. For stable patients, further classification is based on ASA grade, serum C-reactive protein (CRP) levels, and stone size. Patients with ASA ≤ 2, CRP ≤ 200 mg/L, and ureteral stones ≤ 7 mm in size may undergo primary ureteroscopy, as all patients (n = 18) in our series that satisfied these conditions achieved complete stone clearance at primary ureteroscopy without any postoperative complications. Should frank pus be encountered at any point during attempted primary ureteroscopy, we recommend abandoning ureteroscopy, to decompress with a ureteric stent, and pursue delayed stone removal after a period of antibiotic therapy and renal tract decompression, Similarly, we propose that patients with ASA ≥ 2, CRP ≥ 200 mg/L, or stone size > 7 mm should undergo urgent decompression with deferred stone removal, either during the same hospital admission once clinically stable, or on an early outpatient elective operating list. These clinical parameters were all associated with worse clinical outcomes in patients undergoing definitive stone treatment without prior decompression. If complete stone clearance cannot be achieved at primary ureteroscopy, patients should be decompressed with a retrograde stent, with planned early elective ureteroscopy. While this algorithm should not replace clinical judgement, we feel that it may be useful as a guide for urologists when planning further clinical trials. Future randomised studies should focus on refining the criteria for patient selection to determine which individuals are most likely to benefit from primary ureteroscopy and which would be better served by delayed intervention. Additionally, multicentre trials with larger patient populations are needed to validate these findings and to clarify the role of early intervention in high-risk populations.

The non-randomised nature of this study, and its inherent biases, along with the relatively small sample size, and careful patient selection are limitations of this study. Additionally, the small sample size increases the risk of Type II errors, as indicated by post hoc power analyses, which suggests that the study may have been underpowered to detect clinically meaningful differences between groups. Although the findings provide valuable insights, larger studies are needed to confirm the safety and efficacy of early ureteroscopy in this patient population. Moreover, the study was conducted at only two centres, which may limit the generalisability of the results. The institutional protocols and surgeon experience at these centres could influence the outcomes, and future multicentre studies are needed to establish definitive consensus. The allocation of patients into subgroups based on clinical factors, while appropriate, also has an inherent selection bias when comparing these groups.

## Conclusion

In conclusion, this study demonstrates select patients presenting with obstructed infected ureteral stones can safely undergo primary ureteroscopy with definitive stone treatment without an initial period of decompression. Primary ureteroscopy was associated with high stone clearance rates, shorter hospital stays, and low postoperative complication rates. However, delayed intervention remains necessary for patients with significant comorbidities or more complicated clinical presentations. Future studies should focus on optimising patient selection and determining the most appropriate timing for intervention in this high-risk population.
